# *In Vitro* Effects of Concomitant Use of Herbal Preparations on Cytochrome P450s Involved in Clozapine Metabolism

**DOI:** 10.3390/molecules21050597

**Published:** 2016-05-06

**Authors:** Wei Wang, Dan-Dan Tian, Zhang-Jin Zhang

**Affiliations:** School of Chinese Medicine, LKS Faculty of Medicine, the University of Hong Kong, 10 Sassoon Road, Pokfulam, Hong Kong, China; wwei0825@gmail.com (W.W.); tiandd86@hotmail.com (D.-D.T.)

**Keywords:** herb-drug interaction, clozapine, human liver microsomes, recombinant CYPs

## Abstract

Herbal supplements are increasingly used in psychiatric practice. Our epidemiological study has identified several herbal preparations associated with adverse outcomes of antipsychotic therapy. In this study, we evaluated the *in vitro* effects of four herbal preparations—*Radix Rehmanniae* (RR), *Fructus Schisandrae* (FS), *Radix Bupleuri* (RB) and *Fructus Gardeniae* (FG)—on cytochrome P450s (CYPs) involved in the metabolism of clozapine in human liver microsomes (HLMs) and recombinant human cytochrome P450 enzymes (rCYPs). *N*-desmethylclozapine and clozapine *N*-oxide, two major metabolites of clozapine, were measured using high-performance liquid chromatography (HPLC). FG, RR and RB showed negligible inhibitory effects in both *in vitro* systems, with estimated half-maximal inhibitory concentrations (IC_50_) and apparent inhibitory constant values (*K*_i_) greater than 1 mg/mL (raw material), suggesting that minimal metabolic interaction occurs when these preparations are used concomitantly with clozapine. The FS extract affected CYP activity with varying potency; its effect on CYP 3A4-catalyzed clozapine oxidation was relatively strong (*K*_i_: 0.11 mg/mL). Overall, the weak-to-moderate inhibitory effect of FS on *in vitro* clozapine metabolism indicated its potential role in herb-drug interaction in practice.

## 1. Introduction

According to the World Health Organization, traditional medicine meets the primary healthcare needs of 80% of the population in undeveloped regions [1]. As herbal products are obtained from natural sources, they are usually regarded as safe for human consumption. Combinations of herbal and conventional medication are increasingly prescribed to provide synergistic/additive therapeutic efficacy or to reduce side effects induced by synthetic drugs. However, concerns about herb-drug interactions (HDI) have increased along with the growth of polypharmacy. In a previous epidemiological study [2], we found that 36.4% of the schizophrenic patients in our sample had been prescribed herbal medicines in combination with antipsychotics. A small, but significant proportion of these patients experienced more negative clinical consequences than those who received antipsychotics only, suggesting the potential existence of HDI.

Clozapine (CLZ), an antipsychotic medication, is prescribed to a large proportion of schizophrenia patients and is the preferred first-line treatment for schizophrenia in China [3]. It is also the medication most commonly prescribed to refractory-schizophrenia patients worldwide [4]. In this study, we measured the kinetic effects on CLZ metabolism of four herbal medicines: *Radix Rehmanniae* (*Rehmannia glutinosa* Libosch; RR; Di-Huang), *Fructus Schisandrae* (*Schisandra chinensis* Baill; FS; Wu-Wei-Zi), *Radix Bupleuri* (*Bupleurum chinense* DC.; RB; Chai-Hu) and *Fructus Gardeniae* (*Gardenia jasminoides* Ellis; FG; Zhi-Zi). These four medicines were selected due to their extensive clinical use and pharmacological benefits in the treatment of psychotic disorders [5,6,7] and their significant observed correlation with adverse clinical outcomes when concomitantly consumed with antipsychotic agents [2].

Natural herbs and synthetic pharmaceuticals always share the same metabolizing enzymes before excretion from the human body. The cytochrome P450 (CYP) enzyme system is responsible for the biotransformation of more than 90% of the drugs on the market [8]. Most CYP enzymes are found in the liver, which serves as the primary site for the metabolism of psychotropic agents [9]. As previously reported, CLZ is catalyzed to two major metabolites by a group of CYPs, principally CYP 1A2, CYP 3A4, CYP 2D6 and CYP 2C19 [10], at the hepatic level. CYP 1A2 plays a major role in mediating the formation of *N*-demethyl CLZ (norCLZ), while both CYP 2D6 and CYP 2C19 contribute minorly, and CLZ *N*-oxide is primarily catalyzed by CYP 3A4 [11]. Research has shown that the alteration of CYP enzyme activities by inhibitors or inducers plays an important role in the mechanism of HDI [12]. So far, little research was focused on the pharmacokinetic interaction between herbal medicines and antipsychotics. Such HDI becomes critical as psychiatric patients would have a long-period medication, but limited information could be found for guidance to the clinicians. Therefore, characterizing the influence of the above four herbal drugs on the CYP activities involved in CLZ metabolism is of great clinical significance. In this study, we performed kinetic analysis to investigate the effects of RB, RR, FG and FS on the metabolism of clozapine in human liver microsomes (HLMs) and recombinant CYP enzymes (rCYPs).

## 2. Results

### 2.1. Quantitative Analysis of Major Bioactive Constituents of Herbs

A marker-based approach was used to standardize the herbal extracts. Specific HPLC methods based on the previous reports [13,14,15,16] were developed to measure two marker components from each of the four herbal preparations. Before each injection, a mobile phase that was the same as the initial condition of the gradient elution would last for 10 min for equilibrium. The content of the eight bioactive markers is summarized in Table 1. Quantitative analysis revealed that all of the specific constituents were well characterized, with the exception of saikosaponin D from the RB extract, which was found to be below the detection level.

### 2.2. Inhibition Analysis in Pooled HLMs

**Figure 1:**
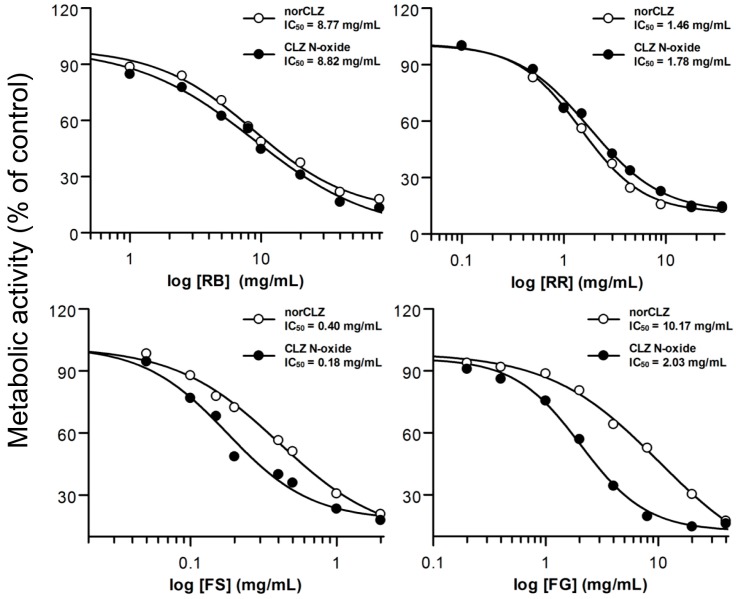
Inhibition of CLZ metabolism in HLMs by various concentrations of *Radix Rehmanniae* (RR), *Fructus Schisandrae* (FS), *Radix Bupleuri* (RB) and *Fructus Gardeniae* (FG). Experiments conducted in triplicate.

The effects of the four herbal extracts on the formation of norCLZ and CLZ *N*-oxide in pooled HLMs were thoroughly examined. Michaelis–Menten constant (*K*_m_) values of 58.1 µM for CLZ demethylation and 34.3 µM for oxidation were obtained from pilot experiments, with maximal-velocity (*V*_max_) values of approximately 687 and 915 pmol/min per milligram of protein, respectively. We then used a substrate concentration of 50 µM CLZ in the following dose-response experiments involving herbs. The IC_50_ values obtained for the four herbal extracts are indicated in Table 2 and Figure 1. The RB and RR preparations were found to have an equivalent inhibitory effect on CLZ demethylation and the formation of oxidative products, with IC_50_ values of approximately 8.8 mg/mL for RB and 1.6 mg/mL for RR. FS exhibited distinct suppression effects on the formation of two metabolites, with IC_50_ values of 0.40 mg/mL for norCLZ and 0.18 mg/mL for CLZ *N*-oxide. FG varied dramatically in its inhibitory effect on the formation of norCLZ (10.17 mg/mL) and CLZ *N*-oxide (2.03 mg/mL).

### 2.3. Inhibition Analysis with Recombinant CYPs

**Figure 2:**
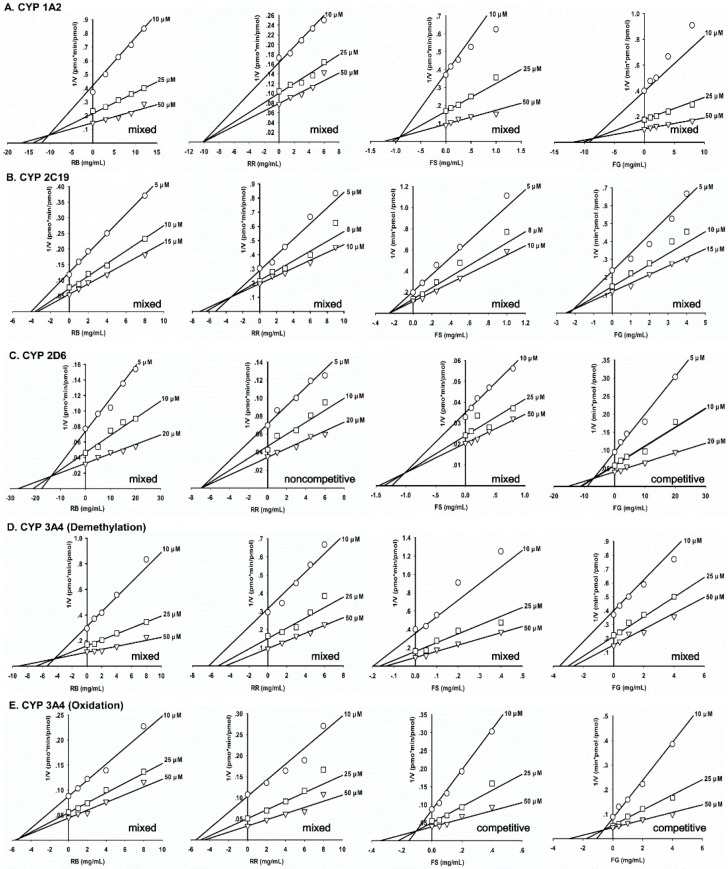
Dixon plot analysis of CLZ demethylation to norCLZ catalyzed by CYP 1A2 (**A**); CYP 2C19 (**B**); CYP 2D6 (**C**); CYP 3A4 (**D**) and CLZ oxidation to CLZ *N*-oxide by CYP 3A4 (**E**) with various concentrations of *Radix Rehmanniae* (RR), *Fructus Schisandrae* (FS), *Radix Bupleuri* (RB) and *Fructus Gardeniae* (FG) at fixed CLZ concentrations.

The inhibitory potency of the four herbs on the CYP isoforms (CYP 1A2, 2C19, 2D6 and 3A4) involved in CLZ metabolism was further examined in rCYPs. The results are summarized in Table 3 and Figure 2. *K*_m_ and *V*_max_ values for the individual isoforms involved in CLZ metabolism are listed. The inhibition modes of the four herbs for individual isoforms are indicated in respective Dixon plots. RR and RB weakly inhibited the formation of both norCLZ and CLZ *N*-oxide by all four of the CYPs, with inhibitory constant (*K*_i_) values of 3.00–10.8 and 3.90–13.8 mg/mL, respectively. FS was found to have a greater inhibitory potential: the herb moderately inhibited CYP activity, with *K*_i_ values ranging from 0.11–1.05 mg/mL. FG weakly suppressed the demethylation of CLZ catalyzed by CYP 1A2, CYP 2C19, CYP 2D6 and CYP 3A4 (*K*_i_: 2.23–8.80 mg/mL), but exhibited a slightly more potent inhibitory effect on the CYP 3A4-mediated formation of CLZ *N*-oxide, with a *K*_i_ of 0.85 mg/mL.

## 3. Discussion

Herbal products are popular worldwide, especially as complementary and alternative forms of medicine. This study investigated the metabolic influence of four commonly-used herbal medicines on clozapine metabolism in HLMs and recombinant CYP enzymes. First, the major constituents of the herbal preparations under study were determined. It is well known that the quality control of herbal medicines relies on the identification and quantification of their chemical constituents, since the contents of bioactive chemicals correlate with herbal pharmacological outcomes and clinical value. In our experiment, two components of each herb were selected as markers to perform a valid and quick detection analysis. The data obtained from the extracts were in line with the findings of previous reports [14,17,18,19]. Saikosaponin D was found to be under the detection limit (0.07 mg/mL), due to the water extraction process. Therefore, the prepared herbal extracts were found to be highly suitable for use in our experimental study.

The results for HLMs, which contain abundant CYP enzymes, are shown in Figure 1 and Table 2. These findings suggest that RB, RR and FG played only a minor role in reducing the formation of CLZ metabolites. However, FS had a weak to moderate suppression effect on CLZ biotransformation; its effect on oxidative metabolism was especially strong. The distinct activities were also shown in the experiment involving FG, whose IC_50_ value for CLZ demethylation was approximately five-times greater than that for oxidation. The explanation may be attributed to the involvement of diverse CYPs in various metabolic pathways. To address this issue, the effects of the herbal extracts were further evaluated by examining the inhibition of individual CYP isoforms, as the “apparent” *K*_i_ value offers a better measure of the interaction of an inhibitor with a particular enzyme [20]. The results were similar to those of the HLM experiments (Figure 2 and Table 3). RB and RR had a negligible influence on all of the CYP isoforms. The FG extract exhibited a very weak inhibitory effect on demethylation induced by the CYP isoforms 1A2, 2C19, 2D6 and 3A4 (*K*_i_ values of 2.23–8.80 mg/mL). However, FG was found to have a greater potential to inhibit CYP 3A4-induced oxidation, with an estimated *K*_i_ value of 0.85 mg/mL. Consistent results were also obtained in the experiments involving FS. CYP 3A4-mediated CLZ oxidation was found to be sensitive to the addition of FS (*K*_i_ value of 0.11 mg/mL), unlike CLZ demethylation via multiple isoforms (*K*_i_ values from 0.21–1.05 mg/mL). Our results are in accordance with those published, finding that CYP 3A4 activity was noticeably inhibited by FG and FS, either extract or their certain components [21,22,23]. In this study, we make the novel proposal that the inhibition of CYP 3A4 activities by FG and FS is metabolic pathway dependent. This partly supports Mooiman’s [24] argument that at least two substrates must be tested in any given *in vitro* study to prevent inter-laboratory differences and confirm the effects of inhibitors on CYP activity.

Although the results of our study suggest that the metabolism of CLZ was moderately inhibited by FS, it is important to note that these *in vitro* findings may not be replicable in a clinical environment. Iwata [25] reported that a variety of constituents of FS exhibited *in vitro* inhibitory effects on CYP 3A4 in human P450 isoforms. In particular, gomisin C was found to be a more potent inhibitor of CYP 3A4 than ketoconazole. In a rat model, Lai [26] and Su [27] confirmed that a single dose of FS inhibited CYP 1A2 and CYP 3A4 and induced CYP 2E1. However, Mu [28] found that the long-term use of FS elevated the expression of drug-metabolizing enzymes, such as CYP 3A and CYP 2C, by activating the xenobiotic orphan nuclear receptor pregnane X receptor, ultimately accelerating the biotransformation of certain drugs. These inconsistent results reflect the complexity of *in vivo* settings for drug metabolism.

To conclude, the relatively high *K*_i_ values obtained for RB, FG and RR suggest that these herbs have little potential for direct pharmacokinetic interaction with CLZ in practice. The adverse clinical outcomes observed should be attributed primarily to other factors, for instance the therapeutic predominance of multi-drug regimens rather than single herbs, suggesting potential herb-herb interaction. FS may affect CLZ blood concentration and increase risk indicated by inhibited CYP enzymes *in vitro*. However, more research is needed to accurately determine the *in vivo* pharmacokinetic effect of FS on CLZ biotransformation.

## 4. Experimental Section

### 4.1. Drugs and Reagents

CLZ (>99.0% purity), norCLZ (>99.0% purity) and CLZ *N*-oxide (>99.0% purity) were obtained from Selleck Chemicals (Houston, TX, USA), Tocris (Bristol, UK) and Enzo Life Sciences, Inc. (Farmingdale, NY, USA), respectively. The bioactive compounds contained in the four herbal medicines, saikosaponin A (>98.0% purity), saikosaponin D (>98.0% purity), geniposide (>98.0% purity), gardenin A (>98.0% purity), catalpol (>98.0% purity), acteoside (>98.0% purity), schisandrin (>98.0% purity) and schisandrol B (>98.0% purity), were all obtained from Shanghai Yuanye Bio-Technology Co. Ltd. (Shanghai, China). Pooled HLM from 25 donors (18 males and 7 females) and recombinant CYP 1A2, CYP 2C19, CYP 2D6 and CYP 3A4 isoforms were purchased from BD Gentest (Woburn, MA, USA). NADPH tetrasodium salt (>95.0% purity) was purchased from Santa Cruz Biotechnology, Inc. (Dallas, TX, USA). High-performance liquid chromatography (HPLC)-grade solvents were supplied by Duksan (Ansan, Korea), and the other analytical reagents used in the study were obtained from Sigma-Aldrich (St. Louis, MO, USA).

### 4.2. Herbal Preparation and Quality Determination

The four herbal medicines under study were prepared using the same protocol. The raw materials for *Radix Rehmanniae* (RR), *Radix Bupleuri* (RB), *Fructus Schisandrae* (FS) and *Fructus Gardeniae* (FG) were supplied and deposited by the pharmacy at the School of Chinese Medicine, the University of Hong Kong. Voucher specimens were identified by Dr. Yan-Bo Zhang at the School of Chinese Medicine, the University of Hong Kong. As recommended in *The Pharmacopoeia of the People’s Republic of China*, water extraction was used to preserve the bioactive constituents [29]. Briefly, the raw materials for each preparation (1.8 kg) were sliced and broiled, then immersed and boiled in a 10-fold volume of distilled water for 2 h. This process was repeated twice, as reported in an earlier study [30]. Next, the solution obtained for each herb was pooled and concentrated to a 1.8-L volume to create a stock solution of 1 g/mL for further use.

To ensure that the herbal preparations were of a high quality and to prevent variation in their composition, all of the extracts were prepared individually from the same batch. The content of the bioactive constituents was measured using reverse-phase HPLC.

### 4.3. Inhibition Assay in HLMs

First, we determined the concentration of each herbal drug required to inhibit the original enzyme activity in HLMs by 50% (IC_50_). CLZ was dissolved in water with 0.05% acetic acid. As reported in a previous study, incubation time and protein concentration were selected to ensure the linear formation of norCLZ and CLZ *N*-oxide [10]. In a pilot study, a *K*_m_ value of approximately 50 µM was obtained for CLZ in HLMs and, thus, chosen as the optimal concentration for IC_50_ determination. In addition, the linear formation of the two metabolites in terms of incubation time was confirmed and 15 min for HLM was found with good linearity. Incubations were performed in triplicate in polypropylene tubes. The typical system contained 100 mM potassium phosphate buffer (pH 7.4) and 50 µg of HLMs in a final volume of 100 µL. CLZ and the individual inhibitors, namely RB, RR, FS and FG, were added to the mixture and placed in a 37 °C water bath for 5 min. Next, the reactions were initiated by adding 1 mM NADPH and terminated by adding 100 µL ice-cold acetonitrile 15 min later. The samples were centrifuged at 12,000× *g* for 10 min, and an aliquot (50 µL) of supernatant was separated for HPLC analysis.

### 4.4. Inhibition Assay with Recombinantly-Expressed Enzymes

The inhibitory effects of individual herbal preparations on specific CYP enzymes were further examined in recombinant P450 isoforms. The incubation mixtures contained CYP isoforms (20 pmol/mL) instead of HLM proteins. The subsequent experimental conditions were similar to those for the HLM mixtures. The substrate, CLZ, was added in serial concentrations from 5–50 µM, in the absence of the herbal extracts and in the presence of the herbal extracts at five serial concentrations from 0.05–20 mg/mL. The reactions lasted for 15 min each, except the reaction involving CYP 2C19, which lasted for 30 min. The samples were centrifuged and stored at −80 °C to enable the measurement of norCLZ and CLZ *N*-oxide.

### 4.5. Measurement of Individual Herbal Bioactive Constituents and CLZ Metabolites

The samples were chromatographically separated on an ACE5 AQ column (5 mm, 4.6 × 250 mm; Advanced Chromatography Technologies Ltd., Aberdeen, UK) using a Waters 626 series HPLC system (Waters, Milford, MA, USA). Different mobile-phase conditions were used to measure the constituents of the four herbal extracts. Details of the separation methods and the absorbance wavelength applied are provided in Table 1. To measure the CLZ metabolites, the analytes were separated in a mobile phase consisting of Solvent A, acetonitrile, and Solvent B, 0.1% (*v*/*v*) acetic acid, in water at a flow rate of 1 mL/min. A gradient elution was used to analyze the CLZ metabolites: 0–18 min, 14%–20% A; 18–30 min, 20%–40% A; 30–35 min, 40%–60% A; 35–40 min, 14% A. The absorbance of norCLZ and CLZ *N*-oxide was measured at 240 nm. The precision of norCLZ and CLZ *N*-oxide was less than 7.0% and 6.3%, respectively. The accuracy of norCLZ and CLZ *N*-oxide was within ±5.9% and ±10.5%. All within the acceptable range. The lower limit of quantification of norCLZ was 0.79 µM and 1.67 µM for CLZ *N*-oxide. The representative HPLC chromatogram is shown in Supplementary Figure S1.

### 4.6. Data Analysis

All of the data are represented as the means of triplicate measurements. The *K*_m_ and *V*_max_ values for CLZ demethylation and oxidation obtained in the HLM experiments were analyzed using Prism 5 (GraphPad Software Inc., La Jolla, CA, USA). GraFit 5 (Erithacus Software Limited, Surrey, UK) was used to calculate the IC_50_ values. The data obtained from the recombinant-enzyme experiments were analyzed using SigmaPlot software (Systat Software Inc., Chicago, IL, USA). The *K*_i_ and the mode of inhibition were estimated and determined by nonlinear regression analysis of metabolite formation, with equations for competitive, noncompetitive, mixed and uncompetitive inhibition, respectively. The goodness of fit was assessed using the lowest Akaike information criterion value. In addition, Dixon plots were visually inspected to verify the mode of inhibition. Cornish-Bowden plots were applied to double check.

## Figures and Tables

**Table 1:** Measurement of bioactive constituents of *Radix Rehmanniae* (RR), *Fructus Schisandrae* (FS), *Radix Bupleuri* (RB) and *Fructus Gardeniae* (FG).

Herb	Mobile Phase	Conditions ^a^	λ_max_ (nm)	Compound	Content (mg/g) ^b^
RR	ACN/0.1% formic acid	0–12 min, 1%–5%; 12–15 min, 5%–10%; 15–18 min, 10%–60%; 18–20 min, 60%–80%; 20–25 min, 80%–1%	210	Catalpol	5.60 ± 0.28
				Acteoside	1.59 ± 0.24
FS	MeOH/H_2_O	0–25 min, 65%	215	Schisandrin	0.65 ± 0.05
				Schisandrol B	1.84 ± 0.37
RB	ACN/0.1% formic acid	0–3 min, 30%–40%; 3–9 min, 40%–60%; 9–20 min, 60%–80%; 20–22 min, 80%–30%	210	Saikosaponin A	0.39 ± 0.05
				Saikosaponin D	-
FG	ACN/0.1% formic acid	0–10 min, 5%–10%; 10–20 min, 10%–50%; 20–22 min, 50%–80%; 22–23 min, 80%; 23–24 min, 80%–5%	240	Geniposide	48.3 ± 12.0
			210	Gardenin A	0.32 ± 0.02

^a^ Chromatography conditions expressed as organic-phase ratio. ^b^ Data expressed in the form of the mean ± standard deviation (SD) mg per g raw materials.

**Table 2:** Potency of inhibitory effects of *Radix Rehmanniae* (RR), *Fructus Schisandrae* (FS), *Radix Bupleuri* (RB) and *Fructus Gardeniae* (FG) on CLZ metabolism in human liver microsomes ^a^.

Metabolites	*K*_m_ (µM)	*V*_max_ (pmol/min/mg Protein)	IC_50_ (mg/mL)			
			RR	FS	RB	FG
norCLZ	58.1 ± 2.46	687 ± 18.8	1.46 ± 0.06	0.40 ± 0.10	8.77 ± 0.60	10.2 ± 2.41
CLZ *N*-oxide	34.3 ± 1.25	915 ± 36.7	1.78 ± 0.05	0.18 ± 0.00	8.82 ± 0.84	2.03 ± 0.16

^a^ Data expressed in the form of the mean ± standard error of mean (SEM) (*n* = 3).

**Table 3:** Potency of the inhibitory effects of *Radix Rehmanniae* (RR), *Fructus Schisandrae* (FS), *Radix Bupleuri* (RB) and *Fructus Gardeniae* (FG) on CLZ metabolism in human recombinant cytochrome P450s ^a^.

CYPs	Metabolites	*K*_m_ (µM)	*V*_max_ (pmol/min/pmol Isoform)	*K*_i_ (mg/mL)			
				RR	FS	RB	FG
1A2	norCLZ	83.9 ±12.1	30.9 ± 3.90	10.8 ± 1.32	0.97 ± 0.12	11.5 ± 1.40	8.80 ± 0.51
2C19	norCLZ	23.8 ± 6.91	33.9 ± 10.6	3.00 ± 0.78	0.21 ± 0.02	3.90 ± 0.52	2.23 ± 0.07
2D6	norCLZ	12.2 ± 0.70	49.3 ± 2.12	6.00 ± 0.47	1.05 ± 0.08	13.8 ± 0.18	7.77 ± 0.59
3A4	norCLZ	66. 5 ± 12.1	21.2 ± 2.84	7.43 ± 0.73	0.22 ± 0.04	5.37 ± 0.73	4.83 ± 0.19
3A4	CLZ *N*-oxide	21.9 ± 4.52	34.9 ± 3.94	5.83 ± 0.61	0.11 ± 0.00	4.47 ± 0.78	0.85 ± 0.14

^a^ Data expressed in the form of the mean ± SEM (*n* = 3).

## Supplementary Materials

Supplementary materials can be accessed at: http://www.mdpi.com/1420-3049/21/5/597/s1.

## Abbreviations

CLZ, clozapine; norCLZ, *N*-desmethylclozapine; CLZ *N*-oxide, clozapine *N*-oxide; CYPs, cytochrome P450s; RR, *Radix Rehmanniae* (Di-Huang); FS, *Fructus Schisandrae* (Wu-Wei-Zi); RB, *Radix Bupleuri* (Chai-Hu); FG, *Fructus Gardeniae* (Zhi-Zi); HPLC, high-performance liquid chromatography; *K*_m_, Michaelis-Menten constant; *V*_max_, maximal-velocity; IC_50_, half-maximal inhibitory concentration; *K*_i_, inhibitory constant.
