# Supplementary material for: In Vitro Effects of Concomitant Use of Herbal Preparations on Cytochrome P450s Involved in Clozapine Metabolism
Source: Molecules. 2016 May 6;21(5):597. doi: 10.3390/molecules21050597 (PMC6273925; doi:10.3390/molecules21050597)
Supplement: Supplementary file 1 [file molecules-21-00597-s001.pdf]

Molecules **2016**, *21*, 597; doi:10.3390/molecules21050597

Supplementary Materials: *In-Vitro* Effects of Concomitant Use of Herbal Preparations on Cytochrome P450s Involved in Clozapine Metabolism

Wei Wang, Dan-Dan Tian and Zhang-Jin Zhang

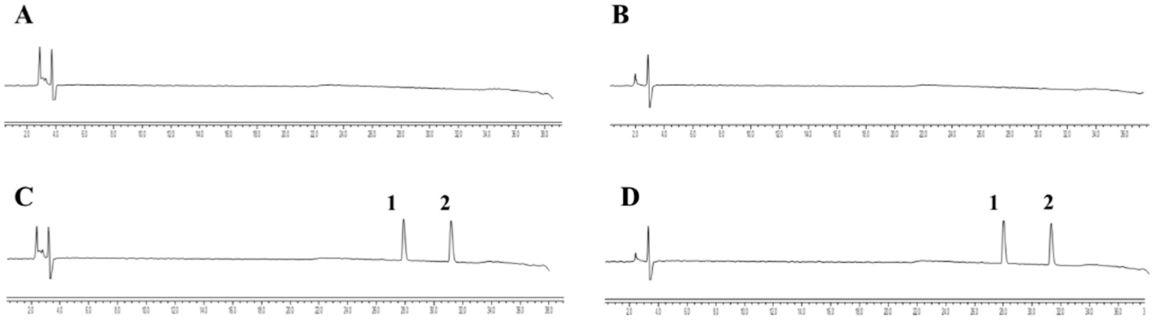

Figure S1. Representative HPLC chromatographic profiles: (**A**) blank-HLM reaction system. (**B**) blank-mixed rCYPs reaction system. (**C**) norCLZ and CLZ *N*-oxide-HLM reaction system. (**D**) norCLZ and CLZ *N*-oxide - mixed rCYPs reaction system. 1, norCLZ; 2, CLZ *N*-oxide.
